# The inflammatory/cancer-related IL-6/STAT3/NF-κB positive feedback loop includes AUF1 and maintains the active state of breast myofibroblasts

**DOI:** 10.18632/oncotarget.9633

**Published:** 2016-05-26

**Authors:** Siti-Fauziah Hendrayani, Bothaina Al-Harbi, Mysoon M. Al-Ansari, Gabriela Silva, Abdelilah Aboussekhra

**Affiliations:** ^1^ Department of Molecular Oncology, King Faisal Specialist Hospital and Research Center, Riyadh, Saudi Arabia; ^2^ Department of Microbiology, Faculty of Science and Medical Studies, King Saud University, Riyadh, Saudi Arabia; ^3^ Current address: Instituto de Biologia Experimental e Tecnológica, Oeiras, Portugal

**Keywords:** IL-6, breast cancer, cancer-associated fibroblasts, positive feedback loop, AUF1

## Abstract

The IL-6/STAT3/NF-κB positive feedback loop links inflammation to cancer and maintains cells at a transformed state. Similarly, cancer-associated myofibroblats remains active even in absence of cancer cells. However, the molecular basis of this sustained active state remains elusive. We have shown here that breast cancer cells and IL-6 persistently activate breast stromal fibroblasts through the stimulation of the positive IL-6/STAT3/NF-κB feedback loop. Transient neutralization of IL-6 in culture inhibited this signaling circuit and reverted myofibrobalsts to a normalized state, suggesting the implication of the IL-6 autocrine feedback loop as well. Importantly, the IL-6/STAT3/NF-κB pro-inflammatory circuit was also active in cancer-associated fibroblasts isolated from breast cancer patients. Transient inhibition of STAT3 by specific siRNA in active fibroblasts persistently reduced the level of the RNA binding protein AUF1, blocked the loop and normalized these cells. Moreover, we present clear evidence that AUF1 is also part of this positive feedback loop. Interestingly, treatment of breast myofibroblasts with caffeine, which has been previously shown to persistently inhibit active breast stromal fibroblasts, blocked the positive feedback loop through potent and sustained inhibition of STAT3, AKT, lin28B and AUF1. These results indicate that the IL-6/STAT3/NF-κB positive feedback loop includes AUF1 and is responsible for the sustained active status of cancer-associated fibroblasts. We have also shown that normalizing myofibroblasts, which could be of great therapeutic value, is possible through the inhibition of this procarcinogenic circuit.

## INTRODUCTION

During the last two decades evidence has accumulated that cancer-associated fibroblasts (CAFs), which constitute a major portion of the reactive breast stromal cells, actively participate in tumor growth, invasion and metastasis [1–3]. Indeed, a large amount of data has emerged showing cancer-promoting function of these cells, which escort tumor cells through all the carcinogenesis steps. This involves many signaling proteins that transmit the message in both directions, allowing cooperative crosstalk between cancer cells and their stroma, which influences the clinical course of the disease [4–6].

Active fibroblasts play similar roles in wound healing and in cancer, which is considered as wound that does not heal [7]. However, while in normal wounds active fibroblasts are transients, CAFs are permanent in tumors. The active status of cancer-associated fibroblasts persists even when these cells are separated from cancer cells and after prolonged cell culture *in vitro* [8, 9]. This could result from genetic and/or epigenetic changes [10]. While epigenetic alterations, especially DNA methylation, were previously reported in breast CAFs, the presence of somatic mutations remains controversial [11–14]. Interestingly, it has been recently shown that natural products such as curcumin and caffeine can persistently normalize the active status of myofibroblasts and inhibit their pro-carcinogenic effects, suggesting that the transactivation of these cells is reversible. While curcumin effect was mediated through the induction of senescence, the molecular mechanisms that underlay the sustained inhibitory effects of caffeine on myofibroblats are still remain unclear [15, 16]. Caffeine increased the expression of the tumor suppressor proteins p16, p21 and p53, and repressed the expression/secretion of various procarcinogenic cytokines such as SDF1, IL-6 and TGF-β1 in active breast fibroblasts [15].

Iliopoulos et al. have shown that transient activation of the Src oncogene induces neoplastic transformation, which is sustained through a positive feedback loop involving IL-6, STAT3, PTEN, NF-κB, Lin28B and let-7b [17, 18]. This loop maintains the epigenetic transformed state of cells for many generations in the absence of the inducing signal. It has been also reported that this epigenetic switch is required for the self-renewing capacity of cancer stem cells [17].

We have recently shown that the aggressive breast cancer MDA-MB-231 cells as well as the interleukin-6 recombinant protein can activate breast stromal fibroblasts in a STAT3-dependent manner [19]. Therefore, we asked in the present study whether this IL-6-related activation of breast stromal fibroblasts is also persistent and the role of the IL-6/STAT3/NF-κB epigenetic feedback loop in maintaining the active status of myofibroblasts.

## RESULTS

### Breast cancer cells and IL-6 mediate sustained activation of breast stromal fibroblasts *in vitro*

**Figure 1:**
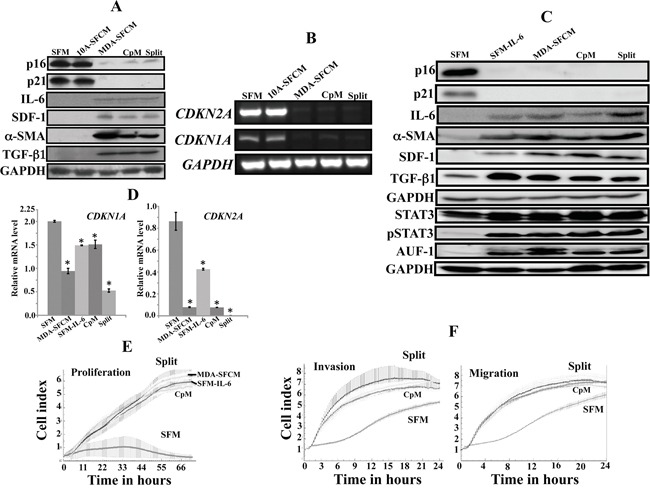
Breast cancer cells and IL-6 persistently activate breast stromal fibroblasts Exponentially growing NBF-6 cells were exposed to SFM, SFM conditioned with MCF-10A cells (10A-SFCM) or MDA-MB-231 cells (MDA-SFCM) for 24 h, and then MDA-SFCM was replaced with complete medium and cells were reincubated for 48 h (CpM), subsequently these cells were split and reincubated for another 48 h (Split). **A.** Cell lysates were prepared and were used for immunoblotting analysis using specific antibodies and GAPDH was used as internal control. **B.** Total RNA was prepared and used for RT-PCR utilizing primers specific for the indicated genes. The amplified products were electrophoresed on 2% ethidium bromide stained agarose gels. These results are typical of at least 3 independent experiments. **C, D.** Cells were exposed for 24 h to SFM, SFM containing IL-6 (35 ng/mL) (SFM-IL6), MDA-SFCM, CpM (48 h) following removal of SFM-IL-6 and also after split of these cells. (C) Cell lysates were prepared and were used for immunoblotting analysis. (D) Total RNA was prepared from the indicated cells, and then the mRNA levels of the indicated genes were assessed by qRT-PCR. Error bars indicate mean +/− SD of three experiments. *: *p* value < 0.05. **E.** Cell proliferation analysis of exponentially growing cells (2.10^4^) using the RTCA-DP xCELLigence System. **F.** The migration and invasion abilities of the indicated cells were assessed for 24 h by the real time RTCA-DP xCELLigence System.

Cancer-associated fibroblasts are permanently active. Since the highly invasive MDA-MB-231 cells can activate breast stromal fibroblasts in a paracrine manner, we sought to investigate whether this *in vitro* activation is also persistent or rather transient. To this end, normal breast fibroblasts (NBF-6) were cultured for 24 h in the presence of serum-free medium (SFM), serum-free conditioned medium (SFCM) from the non-carcinogenic cells MCF-10A (MCF10A-SFCM) or SFCM from MDA-MD-231 cells (MDA-SFCM). Subsequently, MDA-SFCM was replaced with complete medium (CpM) and cells were reincubated for 48 h, and then these cells were split and reincubated for another 48 h (Split). Cell lysates were prepared and the expression level of various markers of active stromal fibroblasts were assessed by immunoblotting using specific antibodies and GAPDH was used as internal control. As expected, MDA-SFCM down-regulated p16 and p21 while it increased the levels of SDF-1, IL-6, α-SMA and TGF-β1 as compared to SFM and MCF10A-SFCM (Figure 1A). This suggested the activation of NBF-6 cells in response to MDA-MB-231 paracrine factors. Interestingly, this effect did not change in the presence of CpM or following cell splitting (Figure 1A). This indicates that the activation of these fibroblasts was sustained upon removal of the paracrine activating factors. The purification of total RNA from the same cells and RT-PCR using specific primers showed that the persistent activating effect of MDA-SFCM was also observed at the mRNA level of the *CDKN1A* and *CDKN2A* genes (Figure 1B). Since IL-6 was also able to activate breast stromal fibroblasts [19], we also tested the persistency of the IL-6-related activation. To address this question, we used the same cells exposed for 24 h to SFM, SFM containing IL-6 (35 ng/mL) (SFM-IL6), MDA-SFCM, CpM (48 h) following removal of SFM containing IL-6 and also after split of these cells. The immunoblotting shows strong down-regulation of p16 and p21 accompanied by important increase in the level of IL-6, SDF-1, α-SMA and TGF-β1 in response to MDA-SFCM and IL-6 as compared to SFM (Figure 1C). This effect was sustained even after the removal of IL-6 and cell splitting (Figure 1C). This indicates that, like MDA-SFCM, IL-6-related activation of breast stromal fibroblasts is also persistent. Since IL-6-dependent activation of breast fibroblasts was mediated through the activation of STAT3 and AUF1 up-regulation [19], we investigated whether these effects were also sustained, and have found persistent increase in the levels of both active STAT3 and AUF1 (Figure 1C). The persistent effect of IL-6 was also observed at the mRNA level of the *CDKN1A* and *CDKN2A* genes following amplification by quantitative RT-PCR (qRT-PCR) (Figure 1D).

To confirm the sustained IL-6-dependent activation of stromal fibroblasts, we tested the proliferation and the migration/invasion abilities of NBF-6 cells exposed to the same media as described above. These experiments were performed using the real time RTCA-DP xCELLigence System. Figure 1E and F shows sustained high proliferation rate and high migration/invasion abilities of NBF-6 cells upon the removal of the inducing factors and split of cells. Together, these results present the first indication that MDA-SFCM and IL-6 persistently activate breast stromal fibroblasts *in vitro*.

### MDA-MB-231 and IL-6 activate the IL-6/STAT3/NF-κB autocrine positive feedback loop in breast stromal fibroblasts

**Figure 2:**
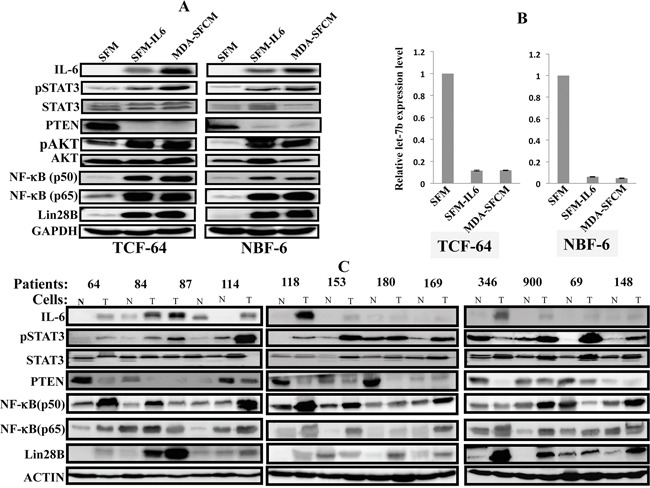
The IL-6/STAT3/NF-κB autocrine positive feedback loop is active in activated fibroblasts *in vitro* and in tumors Exponentially growing cells were exposed for 24 h to SFM, SFM containing IL-6 (35 ng/mL) or MDA-SFCM, and then media were replaced with complete medium and cells were passaged several times. **A.** Cell lysates were prepared and were used for immunoblotting analysis using specific antibodies and GAPDH was used as internal control. **B.** Total RNA was prepared from the indicated cells, and then Let-7b levels were assessed by qRT-PCR. Error bars indicate mean +/− SD of three experiments. **C.** Cell lysates were prepared from breast stromal fibroblasts isolated from tumors (T) and their adjacent histologically normal adjacent tissues (N), and the levels of the indicated proteins were assessed by immunoblotting.

In order to elucidate the molecular mechanism underlying the persistent IL-6-dependent activation of breast stromal fibroblasts, we sought to test the possible activation of the IL-6/STAT3/NF-κB loop in IL-6-activated fibroblasts. To achieve this, we exposed 2 normal breast stromal fibroblasts NBF-6 and TCF-64 for 24 h to SFM, SFM-IL6 and MDA-SFCM, and then these media were replaced with fresh CpM and cells were reincubated and passaged 3 times. Total RNA and proteins were prepared and used to assess the level of Let-7b (qRT-PCR), as well as several other components of the loop (immunobloting). As expected, paracrine IL-6 increased the level of the protein inside breast stromal fibroblasts and activated STAT3 (Figure 2A). Concomitantly, PTEN was down-regulated leading to the activation of AKT and NF-κB, which up-regulated Lin28B (Figure 2A). Additionally, Let-7b was strongly down-regulated in both TCF-64 and NBF-6 cells (Figure 2B). This indicates that paracrine IL-6 induces the positive feedback loop in breast stromal fibroblast cells.

### The IL-6/STAT3/NF-κB positive feedback loop is active in breast cancer-associated fibroblasts

To confirm the active status of the IL-6-related feedback loop in CAFs, we decided to assess the levels of the components of this loop in CAFs as compared to their adjacent tumor counterpart fibroblasts (TCFs) from the same patients. To this end, cell lysates were prepared from 12 pairs CAF/TCF and the expression levels of several elements of the IL-6/STAT3/NF-κB loop were assessed by immunoblotting. Figure 2C shows that IL-6 level is higher in all CAFs as compared to their corresponding TCFs (12 out of 12 pairs), indicating that IL-6 expression increases in active breast myofibroblasts. Consequently, STAT3 was active in 10 out of 12 CAFs as compared to their corresponding TCFs (Figure 2C). Similarly, the levels of the NF-κB subunits p50 and p65 were higher in 8 and 9 out of 12 CAFs as compared to TCFs, respectively (Figure 2C). On the other hand, the tumor suppressor PTEN protein was reduced in 9 out of 12 CAFs relative to TCFs (Figure 2C). Interestingly, Lin28B was up-regulated in 11 out of 12 CAFs as compared to their corresponding TCFs (Figure 2C). These results clearly show that the IL-6/STAT3/NF-κB positive feedback loop is active in breast cancer-associated fibroblasts.

### IL-6 activates the IL-6/STAT3/NF-κB loop and fibroblasts through an autocrine signaling

**Figure 3:**
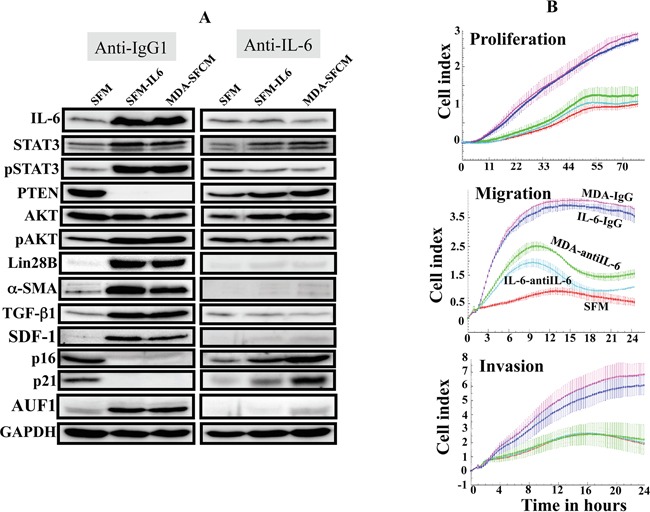
IL-6 activates the IL-6/STAT3/NF-κB feedback loop in an autocrine manner NBF-6 cells were treated as in Figure 2A, and then either anti-IL-6 inhibitory antibody or anti-IgG were added for 24 h, and then cells were subcultured in absence of the inhibitory antibodies. **A.** Cell lysates were prepared and were used for immunoblotting analysis. **B.** Cell proliferation, migration and invasion of the indicated cells were assessed using the RTCA-DP xCELLigence System.

To show the IL-6 autocrine effect in the activation of breast stromal fibroblasts, we cultured activated NBF-6 cells as described above, and then we added directly to cultures either anti-IL-6 neutralizing antibody or IgG for 24 h. Subsequently, cells were recultured in absence of the antibodies for 3 passages. The immunoblotting analysis shows strong increase in the level of IL-6 in control cells pre-activated by IL-6 or MDA-SFCM, but not in cells cultured in the presence of the inhibitory antibody (Figure 3A). This led to the suppression of the related feedback loop (inhibition of STAT3 and AKT) and the suppression of active fibroblasts (strong inhibition of α-SMA, SDF-1 and TGF-β1) (Figure 3A). Interestingly, similar effect was observed for the common regulator of these myofibroblast markers AUF1 [19] (Figure 3A). This suggests that the transient inhibition of secreted IL-6 from active breast stromal fibroblasts persistently suppressed these cells, indicating the presence of an IL-6 autocrine loop, which perpetuates the molecular changes observed in active breast stromal fibroblasts. To confirm this, we assessed the replicative and migratory/invasiveness capacities of active cells challenged with anti-IL-6 antibody as compared to their corresponding controls (IgG) as described above. Interestingly, the presence of extracellular anti-IL-6 antibody strongly reduced the proliferation as well as the migration/invasion abilities of both IL-6- and MDA-SFCM-activated breast fibroblasts (Figure 3B). This shows that the active status of breast myofibroblasts depends on IL-6 autocrine effect, and that transient inhibition of IL-6 led to sustained inhibition of breast stromal fibroblasts.

### Transient inhibition of STAT3 by specific siRNA persistently inhibits the IL-6/STAT3/NF-κB positive feedback loop and inactivates myofibroblast cells

**Figure 4:**
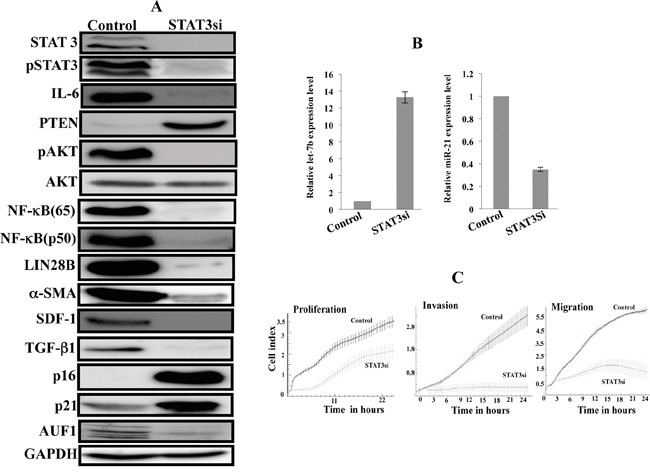
STAT3 inhibition by siRNA persistently inhibits the IL-6/STAT3/NF-κB feedback loop and inactivates myofibroblast cells STAT3 was knocked-down in active breast stromal fibroblasts (CAF-346) with specific siRNA STAT3si (a scrambled sequence was used as control), and then the medium was changed and cells were cultured in siRNA-free medium till confluency and then were splitted several times. **A.** Cell lysates were prepared and used for immunoblotting analysis. **B.** Total RNA was prepared from the indicated cells, and then Let-7b and miR-21 levels were assessed by qRT-PCR. Error bars indicate mean +/− SD of three experiments. **C.** Cell proliferation, migration and invasion of the indicated cells were assessed using the RTCA-DP xCELLigence System.

To show the persistent effect of the active IL-6/STAT3/NF-κB positive feedback loop on breast stromal fibroblasts, we knocked-down STAT3 in active breast stromal fibroblasts (CAF-346) with specific siRNA (a scrambled sequence was used as control). Subsequently, the medium was changed and cells were cultured in siRNA-free medium till confluency, and then were splitted 5 times. Cell lysates were prepared and used for immunoblotting analysis. Figure 4A shows that the levels of STAT3 and the phosphorylated form of the protein remained reduced in absence of the inhibitor. Similarly, the expression levels of all the components of the loop were remained affected by the initial STAT3 down-regulation (Figure 4A). This suggested the persistent inhibition of the positive loop. To confirm this, we checked the levels of both miRNAs let-7b and miR21, and have found that while the first one remained up-regulated the second one, which is a direct target of STAT3, was persistently up-regulated in STAT3siRNA-treated cells (Figure 4B).

Next, we checked the active status of these fibroblasts by assessing the level of myofibroblast markers. Figure 4A shows that the 4 major markers of myofibroblasts (α-SMA, SDF-1, TGF-β1 and IL-6) were remained down-regulated while p16 and p21 were remained up-regulated. Interestingly, the STAT3 down-regulation persistently reduced the level of the major regulator of all these genes (AUF1), which plays key roles in the activation of breast stromal fibroblasts [19]. Furthermore, we have shown that transient STAT3 inhibition persistently reduced the proliferation rate as well as the migration/invasion capacities of active breast stromal fibroblasts (Figure 4C). This clearly shows that transient inhibition of STAT3 led to permanent inactivation of the protein, through the inactivation of the positive feedback loop and the consequent sustained transdifferentiation of breast myofibrobalsts into inactive fibroblasts.

### AUF1 is part of the IL-6/STAT3/NF-κB autocrine positive feedback loop

**Figure 5:**
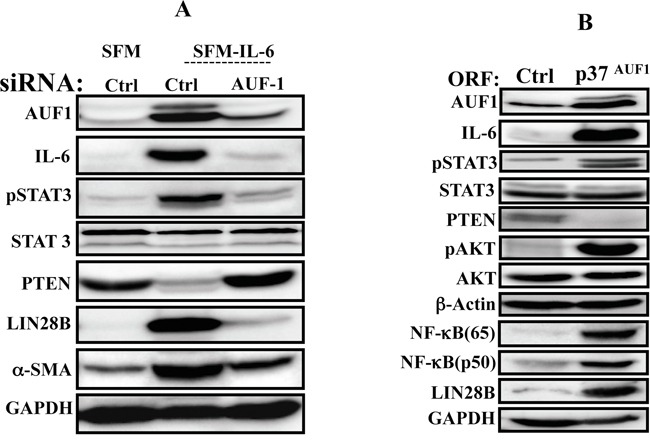
AUF1 is part of the IL-6/STAT3/NF-κB autocrine positive feedback loop **A.** NBF-6 cells were transfected with specific AUF1-siRNA or a scrambled sequence (Ctrl) and then cells were exposed to SFM or SFM containing IL-6 (35 ng/mL) for 24 h. Cell lysates were prepared and used for immunoblotting analysis. **B.** NBF-6 cells were infected with lentivirus based vectors either empty (Ctrl) or bearing p37^AUF1^-ORF, and then the level of the indicated proteins were assessed by immunoblotting.

Since AUF1 is a positive regulator of IL-6 and plays important role in breast stromal fibroblast activation [19], we sought to investigate the possible involvement of this protein in the IL-6-dependent activation of the positive feedback loop. To this end, AUF1 was knocked-down in NBF-6 cells using specific siRNA, and scrambled siRNA was used as control. To assess IL-6 effect, cells were incubated for 24 h in the presence of SFM or SFM containing IL-6. Interestingly, AUF1 knock-down suppressed IL-6-dependent effects on the members of the IL-6/STAT3/NF-κB positive feedback loop (Figure 5A). Indeed, IL-6 treatment of AUF1-deficient cells did not lead to STAT3 activation nor lin28B up-regulation as compared to control cells (Figure 5A). This suggests that AUF1 is a key player in the IL-6-dependent activation of the IL-6/STAT3/NF-κB loop. To confirm this, AUF1 was up-regulated by ectopic expression of the p37^AUF1^ isoform in NBF-6 cells, and then the status of the major components of the loop was evaluated by immunostaining. Figure 5B shows that the increase in the AUF1 level up-regulated IL-6, activated STAT3 and AKT, and induced Lin28B, which indicates the activation of the IL-6/STAT3/NF-κB circuit (Figure 5B). Together, these results provide evidence for the first time that AUF1 participates in the positive feedback loop.

### Caffeine persistently blocks the IL-6/STAT3/NF-κB autocrine positive feedback loop

**Figure 6:**
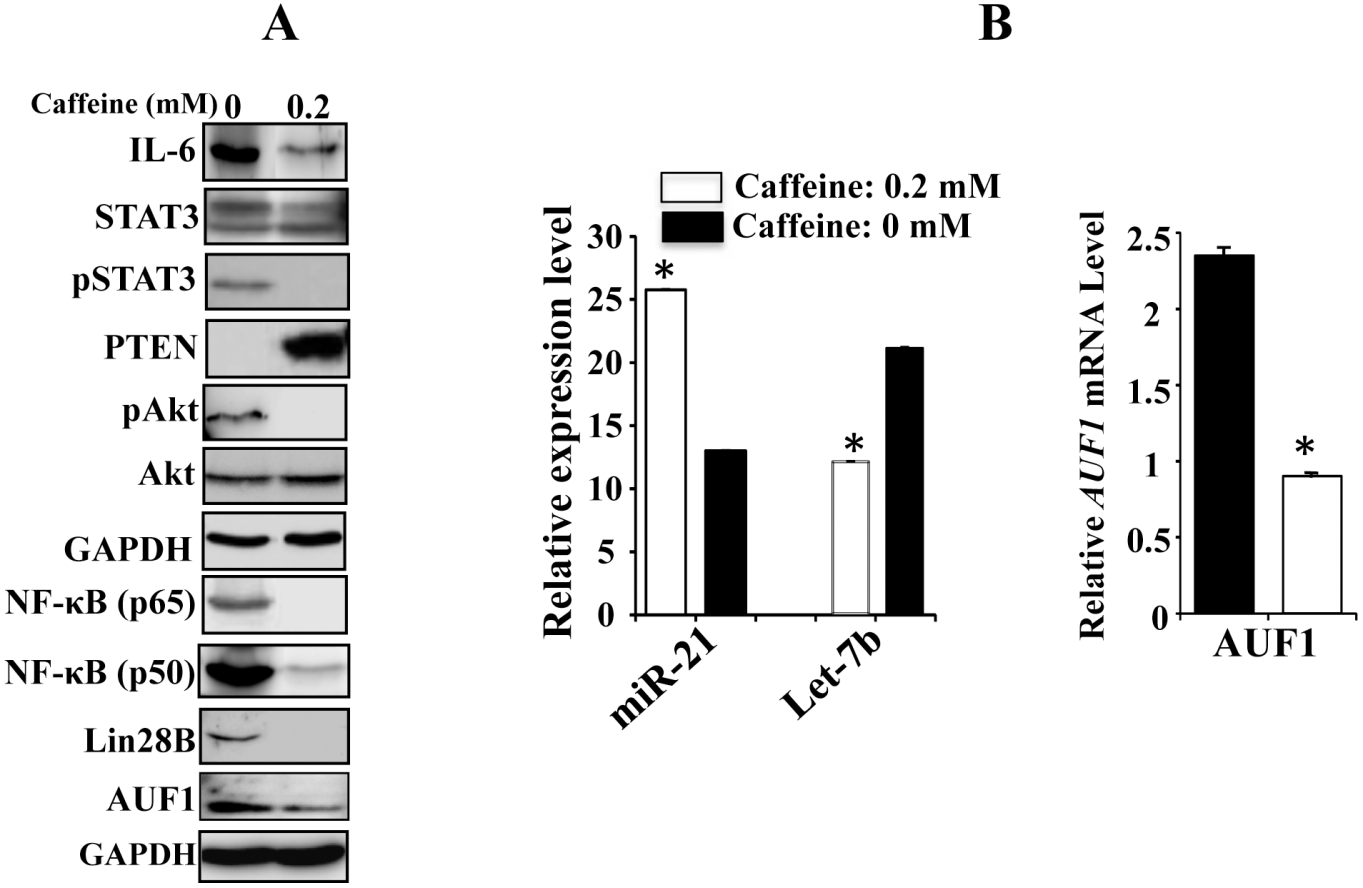
Caffeine persistently blocks the IL-6/STAT3/NF-κB feedback loop CAF-180 cells were either sham-treated (0) or challenged with caffeine (0.2 mM, for 1 hr), and then medium was changed and cells were washed twice with PBS before being reincubated in caffeine-free medium for 24 hr. **A.** Cell lysates were prepared and the levels of various proteins were assessed by immunoblotting. **B.** Total RNA was prepared, and then the levels of Let-7b and miR-21 as well as the AUF1 mRNA were assessed by qRT-PCR. Error bars indicate mean +/− SD of three experiments, *: *p* value < 0.0001.

We have recently shown that caffeine mediates sustained inactivation of breast cancer-associated myofibroblasts [15]. Therefore, we sought to investigate whether this sustained effect is mediated through the inhibition of the IL-6/STAT3/NF-κB loop. To this end, CAF-180 cells were either sham-treated or challenged with caffeine (0.2 mM) for 1h, and then caffeine-containing medium was replaced with caffeine-free medium for 24 h. Subsequently, cell lysates were prepared and used for immunoblotting analysis. Figure 6A shows caffeine-dependent strong inhibition of the three major components of the loop IL-6, NF-κB (p50 and p65), Lin28B and also AUF1. Similarly, caffeine had potent inhibitory effects on the active forms of Akt and STAT3 while it up-regulated PTEN (Figure 6A). To confirm the persistent inhibitory effect of caffeine on the loop we also tested the effect of caffeine on Let-7b and miR-21 by qRT-PCR in CAF-180 cells. Figure 6B shows caffeine-dependent down-regulation of the STAT3 target miR-21, and up-regulation of the Lin28B target Let-7b in these CAF cells. The mRNA level of AUF1, another target of STAT3, was also persistently reduced in response to caffeine (Figure 6B). This clearly shows that caffeine blocks the IL-6/STAT3/NF-κB positive feedback loop, which explains its observed sustained effect on active breast stromal fibroblasts [15].

## DISCUSSION

**Figure 7:**
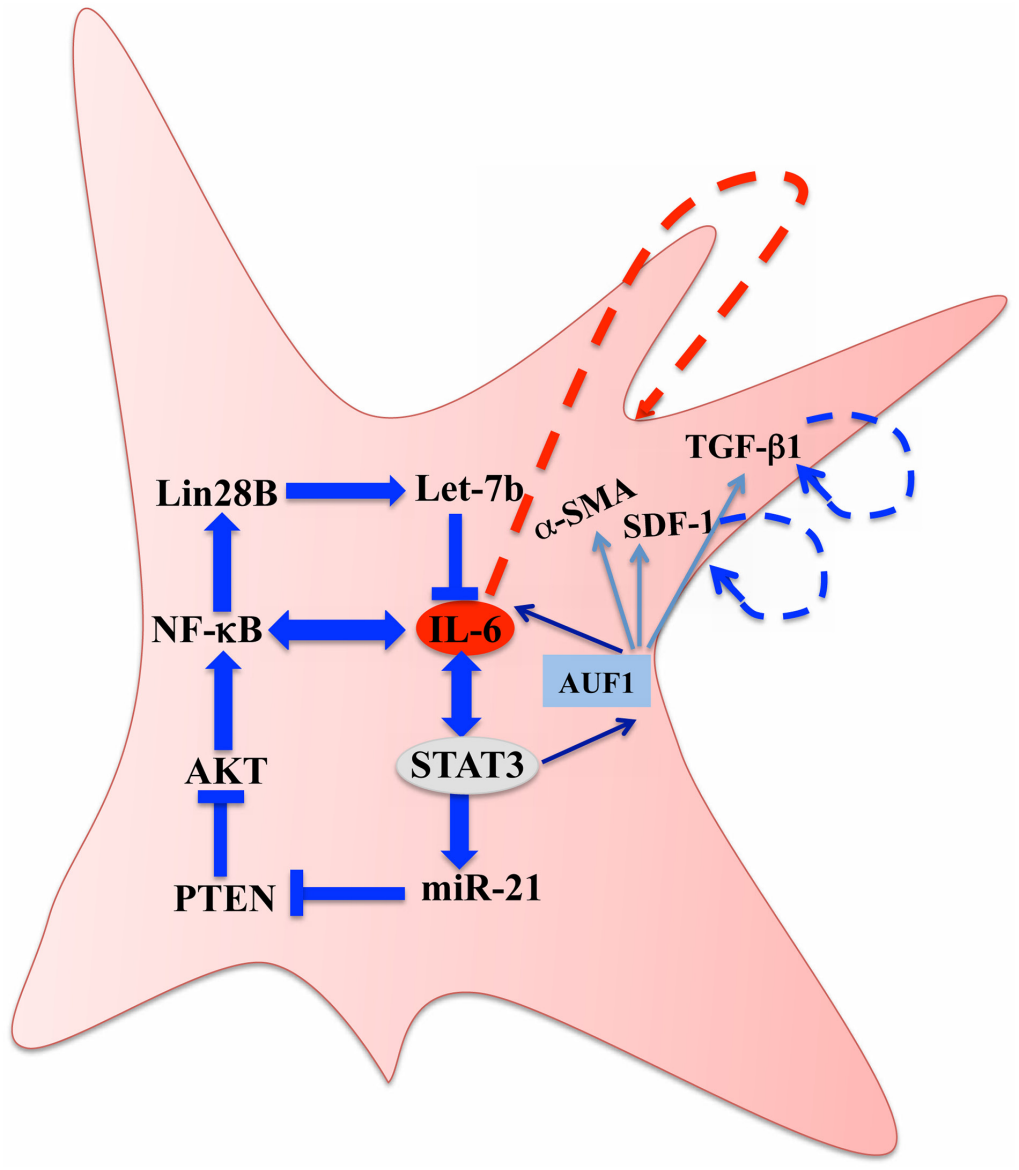
Schematic representation of the IL-6/STAT3/NF-κB feedback loop, and implication of AUF1 in the activation of breast stromal fibroblasts See text for more details.

After showing that IL-6 can transform breast stromal fibroblasts to myofibroblasts [19], we have demonstrated in the present report that this effect is sustained due to the activation of the inflammatory/cancer-related IL-6/STAT3/NF-κB positive feedback loop, which has been previously shown to play crucial roles in cell transformation and tumorigenesis through the induction of an epigenetic switch [17, 18]. This switch maintained the active phenotype of breast stromal fibroblasts in absence of the initiating event. Indeed, STAT3 and AKT remained phosphorylated/active and cells expressed high levels of α-SMA and were highly proliferative and invasive in absence of IL-6, the inducing signal. In addition, we have shown that AUF1 is part of this loop and plays a major role in the transmission of the IL-6 signaling. AUF1, a direct target of STAT3, is a positive regulator of IL-6 as well as other myofibroblast markers namely, α-SMA, SDF-1 and TGF-β1 [19]. We have shown that AUF1 remained up-regulated in response to transient IL-6 treatment (Figure 1C). On the other hand, the AUF1 level remained reduced for several passages upon transient inhibition of STAT3 (Figure 4A). This suggests that AUF1 mediates the positive feedback loop-dependent activation of breast stromal fibroblasts (Figure 7).

In addition, we have shown the presence of a positive feedback loop in which secreted IL-6 increases its own expression, in an autocrine manner. This indicates that IL-6 can drive and maintain stromal fibroblasts in an activated state. This can also explain the active status of cancer-associated fibroblasts in culture in absence of cancer cells. We have also shown that the IL-6/STAT3/NF-κB positive feedback loop is active in 12 CAFs as compared to their respective TCFs isolated form the same patients and cultured simultaneously *in vitro*. This shows that the epigenetic switch, which persistently activates breast stromal fibroblasts, takes place in tumors wherein fibroblasts are in direct contact with cancer cells. Notably, we have previously shown that AUF1 is also highly expressed in most CAFs as compared to their respective TCFs [20], which further shows the role of AUF1 in maintaining the active status of stromal fibroblasts in response to the IL-6-related epigenetic switch.

Importantly, we have found Lin28B, which acts mainly as an oncogene, highly expressed in 11 out of 12 CAFs (91.6%) as compared to their respective TCFs. This suggests that Lin28B up-regulation may play a major role in the activation of breast stromal fibroblasts. Lin28B level is silenced in adult somatic cells but its highly expressed in embryonic stem cells and in different types of tumors [21, 22]. Several lines of evidence indicate that Lin28B is an important regulator of growth and metabolism in stem cells and is critical for the formation of cancer stem cells, and contributes to tumor aggressiveness and spread [21, 23, 24]. Thereby, it is also plausible that Lin28B contributes to these pro-metastatic functions from stromal fibroblasts in a non-cell-autonomous fashion. These possibilities are currently under investigation. In addition to lin28B, we present also the first indication that STAT3 is phosphorylated, IL-6 as well as the NF-kB sub-units (p50 and p65) are up-regulated, while PTEN is repressed in most CAFs as compared to their counterparts TCFs. IL-6 is a pleiotropic cytokine, which plays important roles in the pathophysiology of cancer [25]. Breast cancer tissues express high levels of IL-6 as compared with matched normal tissues and these levels increase with tumor grade [26]. Furthermore, elevated levels of serum IL-6 correlated with poor prognosis of breast cancer patients [27]. These pro-carcinogenic IL-6 effects could be mediated, at least in part, through the activation of NF-κB and STAT3. Indeed, phosphorylated STAT3 is expressed in about 40% of all breast cancers [28, 29], and its constitutive phosphorylation was associated with trastuzumab resistance in HER2-positive breast cancers [30]. Regarding PTEN, it has been shown that the ablation of this tumor suppressor gene in mammary stromal fibroblasts of mice enhanced epithelial tumors [31]. However, the potential prognostic role of these genes in stromal fibroblasts is still not defined.

The active IL-6/STAT3/NF-κB positive feedback loop in CAFs from breast cancer patients shows an important role of this epigenetic switch in sustaining the activated phenotype of CAFs in the absence of cancer cells. This indicates the possible targeting of active CAF cells through inhibiting the IL-6/STAT3/NF-κB positive feedback loop. This could allow sort of normalization of active stromal fibroblasts, which may have great anti-cancer effects. In fact, we have shown that caffeine, which mediates sustained inactivation of CAF cells [15], inhibits the positive feedback loop. This provides clear explanation for the persistent inhibitory effect of caffeine on CAF cells [15]. This indicates that caffeine could be of great therapeutic value via targeting the carcinogenic and metastatic Lin28B/Let-7b axis in active breast stromal fibroblasts and also inhibits the link between inflammation and cancer. Indeed, a clear association was observed between coffee consumption and inflammation. This association was positive with adiponectine but negative with leptin, which is in favor of tumor suppression [32]. Furthermore, it has been recently shown that increasing coffee consumption is associated with significantly smaller invasive breast tumor sizes, a lower proportion of ER-positive tumors, and improved disease-free survival among tamoxifen-treated women with ER-positive breast cancer. The authors have also shown dose-dependent and complete inhibition of AKT phosphorylation in both MCF-7 and MDA-MB-231 cells [33]. This indicates that caffeine can target both cancer cells and their stromal fibroblasts for a highly efficient treatment of breast cancer and also to prevent tumor growth as well as recurrence.

Like caffeine, inhibition of the positive feedback loop by transient STAT3 down-regulation utilizing specific siRNA, or specific inhibition of IL-6 in culture medium, persistently inactivated the loop and CAF cells. This indicates that inactivating the positive feedback loop, which could bring the pro-carcinogenic myofibroblasts back to a normal tumor suppressive phenotype, could be achieved at different levels and with various biological systems. Therefore, targeting the IL-6/STAT3 pathway in both tumor cells and their stromal fibroblasts could present great therapeutic approach for more efficient eradication of tumors.

## MATERIALS AND METHODS

### Cells, cell culture and chemicals

Breast fibroblast cells NBF-6, TCF-64, CAF-180 and CAF-346 were obtained and used as previously described [8]. MDA-MB-231 and MCF-10A cells were obtained from ATCC and were authenticated at ATCC before purchase by their standard short tandem repeat DNA typing methodology, and were routinely tested for the presence of the relevant markers, and were cultured following the instructions of the company. All supplements were obtained from Sigma (Saint Louis, MO, USA) except for antibiotic and antimycotic solutions, which were obtained from Gibco (Grand Island, NY, USA). Human IL-6 recombinant protein (hBA-184) (Santa Cruz, CA). The anti-IL-6 monoclonal antibody (6708.11) was purchased from Sigma-Aldrich, USA.

### Cellular lysate preparation and immunoblotting

This has been performed as previously described [34]. Antibodies directed against alpha smooth muscle actin (α-SMA), transforming growth factor beta 1 (TGF-β1, 2AR2), Stromal-derived factor-1 (SDF-1), AUF1 (ab50692) and IL-6 were purchased from Abcam (Cambridge, MA); Lin28B (D4H1), NF-kB (p50), NF-kB (p65) (D14E12), AKT (C73H10), p-AKT (Thr308), STAT3 and pSTAT3-Tyr705 (D3A7) from Cell Signaling (Danvers, MA); p16^INK4a^ from BD Biosciences (San Jose, CA); p21 (F-5), PTEN (A2B1) and Glyceraldehydes-3-phosphate dehydrogenase (GAPDH, FL-335) were purchased from Santa Cruz (Santa Cruz, CA).

### RNA purification, RT-PCR and qRT-PCR

Total RNA was purified using the TRI reagent (Sigma) according to the manufacturer's instructions, and was treated with RNase-free DNase before cDNA synthesis using the RT-PCR Kit (Clontech, USA) for both miRNAs and mRNAs. For RT-PCR, cDNA was amplified using the Platinum^®^ *Taq* DNA Polymerase (Invitrogen). After electrophoresis on ethidium bromide stained 2% agarose gels, the intensity of the PCR products was determined with the Quantity One program (Bio-RAD) and was normalized against *GAPDH*. For quantitative RT-PCR (qRT-PCR), the RT^2^ Real-Time^™^ SYBR Green qPCR mastermix (Roche, Germany) was used and the amplifications were performed utilizing the light cycler 480 (Roche, germany). The melting-curve data were collected to check PCR specificity, and the amount of PCR products was measured by threshold cycle (Ct) values and the relative ratio of specific genes to *GAPDH* (or U6 for mature miRNAs) for each sample was then calculated. The respective primers are:

*CDKN2A*:5′-GAGGCCGATCCAGGTCATGA-3′ and 5′-GCACGGGTCGGGTGAGA-3′;

*CDKN1A*: 5′-GAGACTCTCAGGGTCGAAAACG-3′ and 5′-GA TTAGGGCTTCCTCTTGGAGAA-3′;

Mature Let-7b: 5′-TGAGGTAGTAGGTTGTGTGGTT-3′

Mature miR-21: 5′-TAGCTTATCAGACTGATGTTGA-3′

### siRNA transfection

STAT3-siRNA and control–siRNA were obtained from QIAGEN, USA. AUF1-siRNA (pSILENCER-AUF15), which targets all four AUF1 isoforms [35], was a generous gift from Dr. Gorospe. The transfections were carried out using the RNAi Fect reagent (QIAGEN) as recommended by the manufacturer.

### Viral infection

Lentivirus based vectors bearing p37^AUF1^-ORF and the control (Origene) were used to prepare the lentiviral supernatant from 293FT cells. Lentiviral supernatants were collected 48 h post-transfection, filtered and used for infection. 24 h later, media were replaced with complete medium and cells were grown for 3 days.

### Cell migration, invasion and proliferation

These assays were performed in a real-time and label-free manner using the xCELLigence RTCA technology (Roche, Germany) that measures impedance changes in a meshwork of interdigitated gold microelectrodes located at the bottom well (E-plate) or at the bottom side of a micro-porous membrane (CIM plate 16). Cell migration and invasion were assessed as per manufacturer's instructions. In brief, 2×10^4^ cells in serum-free medium were added to the upper wells of the CIM-plate with thin layer of matrigel basement membrane matrix (for invasion) or without (for migration), and a complete media was added to the lower chamber wells used as a chemo-attractant. Subsequently, the plates were incubated in the RTCA for 24 h and the impedance value of each well was automatically monitored by the xCELLigence system and expressed as a Cell index value which represents cell status based on the measured electrical impedance change divided by a background value. Each assay was performed in triplicate.

For the proliferation assay, exponentially growing cells (2×10^4^) were seeded in E-plate with complete medium as per manufacturer's instruction. All data were recorded and analyzed by the RTCA software. Cell Index (CI) was used to measure the change in the electrical impedance divided by the background value to represent cell status. Each assay was performed in triplicate.

### Conditioned media

Cells were cultured in medium without serum for 24 h, and then media were collected and briefly centrifuged. The resulting supernatants were used either immediately or were frozen at −80°C until needed.

### Statistical analysis

Statistical analysis was performed by student's t-test and *P* values of 0.05 and less were considered as statistically significant.
